# Supplementary material for: MCH-R1 Antagonist GPS18169, a Pseudopeptide, Is a Peripheral Anti-Obesity Agent in Mice
Source: Molecules. 2021 Feb 27;26(5):1291. doi: 10.3390/molecules26051291 (PMC7957705; doi:10.3390/molecules26051291)
Supplement: Supplementary file 1 [file molecules-26-01291-s001.pdf]

MCH-R1 antagonist GPS18169, a pseudopeptide, is a peripheral anti-obesity agent in mice.

Jean A. Boutin[1,8], Magali Jullian[2], Lukasz Frankiewicz[2,9], Mathieu Galibert[2], Philippe Gloanec[3], Thierry Le Diguarher[4], Philippe Dupuis[5], Amber Ko[6], Laurent Ripoll[1], Marc Bertrand[4], Anne Pecquery[5], Gilles Ferry[7], Karine Puget[2]

[1] Institut de Recherches Internationales Servier, 92284 Suresnes, France ja.boutin.pro@gmail.com (J.A.B.) laurent.ripoll@servier.com (LR)

[2] Genepep SA, 34430 Saint Jean de Vedas, France magali.jullian@genepep.com (M.J.) l.frankiewicz@pracowniacms.pl (L.F.) mathieu.galibert@genepep.com (M.G.) karine.puget@genepep.com (K.P.)

[3] Institut de Recherches Servier, 92150 Suresnes, France phgloanec@gmail.com (Ph. G .)

[4] Technologie Servier, 45520 Gidy, France thierry.le-diguarher@servier.com (T.L.D.) m.bertrand@servier.com (M.B.)

[5] Eurofins Discovery, 86600 Celle l'Evescault, France PhilippeDupuis@eurofins.com (Ph.D.) AnnePecquery@eurofins.com (A.P.)

[6] Eurofins Discovery, 24891 New Taipei City, Taiwan AmberKo@eurofins.com (A.K.)

[7] Institut de Recherches Servier, 78290 Croissy-sur-Seine, France gilles.ferry@servier.com (G.F.)

[8] current affiliation: PHARMADEV (Pharmacochimie et biologie pour le développement), Faculté de Pharmacie, 31000 Toulouse, France

[9] current affiliation: Gyros Protein Technologies AB, 751 83 Uppsala, Sweden

Supplementary Table S1: Characteristics of the peptides and pseudopeptides synthesized for the present study

Code	"N-terminus"	sequence	C-terminus	Bridge	Purity	Experimental mass	Theoretical mass
11371	pGua	[Cys-Met-Leu-Arg-Tyr-Arg-Pro-Cys]-Trp	OH	S-S bridge C1-C8	95,17	1385,92	1386,71
11372	H	Arg-[Cys-Met-Leu-Arg-Tyr-Arg-Pro-Cys]-Trp	OH	S-S bridge C2-C9	95,32	1381,16	1381,72
11373	pGua	[Cys-Met-Leu-Arg-Val-Arg-Pro-Cys]-Trp	OH	S-S bridge C1-C8	95,73	1322,08	1322,67
11374	pGua	[Cys-Met-Leu-Arg-Val-Tyr-hArg-Pro-Cys]-Trp	OH	S-S bridge C1-C9	95,18	1499,06	1499,8
11375	pGua	[Cys-Met-Leu-Arg-Val-Tyr-dArg-Pro-Cys]-Trp	OH	S-S bridge C1-C9	95,29	1485,06	1485,8
11376	pGua	[Cys-Met-Leu-Arg-Val-Ala-hArg-Pro-Cys]-Trp	OH	S-S bridge C1-C9	95,21	1407,22	1407,74
11377	pGua	[Cys-Met-Leu-Val-Tyr-Arg-Pro-Cys]-Trp	OH	S-S bridge C1-C8	97,01	1328,94	1329,6
11378	pGua	[Cys-Met-Leu-Ala-Val-Ala-hArg-Pro-Cys]-Trp	OH	S-S bridge C1-C9	95,16	1322,62	1322,63
11379	pGua	[Cys-Met-Leu-Ala-Ala-hArg-Pro-Cys]-Trp	OH	S-S bridge C1-C8	95,22	1223,7	1223,5
11380	pGua	[Cys-Met-Leu-Arg-Val-Alva-Pro-Cys]-Trp	OH	S-S bridge C1-C8	95,12	1264,96	1265,6
11381	pGua	[Cys-Met-Leu-Arg-Val-2-Ala-bu-Pro-Cys]-Trp	OH	S-S bridge C1-C8	95,43	1250,96	1251,6
11398	pGua	[Asp-Met-Leu-Arg-Val-Tyr-Arg-Pro-Dap]-Trp	OH	Lactam bridge D1-Dap9	98,83	1464,38	1464,72
11399	pGua	[Asp-Met-Leu-Arg-Tyr-Arg-Pro-Dap]-Trp	OH	Lactam bridge, D1-Dap9	96,73	1365,03	1365,66
11400	pGua	[Asp-Met-Leu-Arg-Val-Arg-Pro-Dap]-Trp	OH	Lactam bridge, D1-Dap8	95,82	1301,1	1301,54
11401	pGua	[Asp-Met-Leu-Arg-Val-Arg-Pro-Dab]-Trp	OH	Lactam bridge D1-Dab8	94,78	1315,21	1315,57
11402	pGua	[Asp-Met-Leu-Arg-Val-Arg-Pro-Orn]-Trp	OH	Lactam bridge D1-Orn8	95,37	1329,11	1329,6
11403	pGua	[Asp-Met-Leu-Arg-Val-Tyr-hArg-Pro-Dap]-Trp	OH	Lactam bridge D1-Dap9	95,27	1478,42	1478,74
11404	pGua	[Asp-Met-Leu-Arg-Val-Tyr-dArg-Pro-Dap]-Trp	OH	Lactam bridge D1-Dap9	96,94	1464,43	1464,72
11405	pGua	[Asp-Met-Leu-Arg-Val-Ala-hArg-Pro-Dap]-Trp	OH	Lactam bridge D1-Dap9	96,06	1386,18	1386,65
11406	pGua	[Asp-Met-Leu-Val-Tyr-Arg-Pro-Dap]-Trp	OH	Lactam bridge D1-Dap8	95,28	1308,14	1308,53
11408	pGua	[Asp-Met-Leu-Ala-Ala-hArg-Pro-Dap]-Trp	OH	Lactam bridge D1-Dap8	95,48	1201,98	1202,4
11410	pGua	[Asp-Met-Leu-Arg-Val-2Abu-Pro-Dap]-Trp	OH	Lactam bridge D1-Dap8	95	1230,13	1230,46
12731	pGua	[Asp-Met-Leu-Arg-Val-Arg-Pro-Lys]-Trp	OH	Lactam bridge D1-K8	89,32	1343,32	1343,6

12732	pGua	[Glu-Met-Leu-Arg-Val-Arg-Pro-Dap]-Trp	OH	Lactam bridge E1-Dap8	91,89	1315,6	1315,57
12733	pGua	[Glu-Met-Leu-Arg-Val-Arg-Pro-Dab]-Trp	OH	Lactam bridge E1-Dab8	95,93	1329,01	1329,6
12734	pGua	[Asp-Met-Leu-Gly-Arg-Val-Arg-Pro-Orn]-Trp	OH	Lactam bridge D1-Orn8	90,83	1385,84	1386,4
12735	pGua	[Asp-MeCys-Leu-Arg-Val-Arg-Pro-Orn]-Trp	OH	Lactam bridge D1-Orn8	96,41	1315,02	1315,6
12736	pGua	[Asp-bhMet-Leu-Arg-Val-Arg-Pro-Orn]-Trp	OH	Lactam bridge D1-Orn8	97,75	1343,04	1343,6
12737	pGua	[Asp-Eth-Leu-Arg-Val-Arg-Pro-Orn]-Trp	OH	Lactam bridge D1-Orn8	91,71	1343,02	1343,6
12738	pGua	[Asp-Buth-Leu-Arg-Val-Arg-Pro-Orn]-Trp	OH	Lactam bridge D1-Orn8	92,72	1371,05	1371,7
12739	pGua	[Asp-SeMet-Leu-Arg-Val-Arg-Pro-Orn]-Trp	OH	Lactam bridge D1-Orn8	95,76	1376,3	1376,6
12740	pGua	[Pra-Met-Leu-Arg-Val-Arg-Pro-Aha]-Trp	OH	Triazole bridge	90,32	1339,2	1339,6
12741	pGua	[Aha-Met-Leu-Arg-Val-Arg-Pro-Pra]-Trp	OH	Triazole bridge	90,42	1339,1	1339,6
12742	ArgBzi	[Asp-Met-Leu-Arg-Val-Arg-Pro-Orn]-Trp	OH	Lactam bridge D1-Orn8	91,62	1440,1	1440,74
12743	pGua	[Orn-Met-Leu-Arg-Val-Arg-Pro-Asp]-Trp	OH	Lactam bridge Orn1-D8	91,18	1329,08	1329,6
12744	pGua	[Glu-Met-Leu-Arg-Val-Arg-Pro-Orn]-Trp	OH	Lactam bridge E1-Orn8	91,63	1343,18	1343,62
12745	pGua	[Orn-Met-Leu-Arg-Val-Arg-Pro-Glu]-Trp	OH	Lactam bridge Orn1-E8	90,43	1343,12	1343,62
12746	pGua	[Glu-Met-Leu-Arg-Val-Arg-Pro-Lys]-Trp	OH	Lactam bridge E1-K8	90,18	1357,2	1357,65
12748	pGua	[Asp-Met-dhLeu-Arg-Val-Arg-Pro-Orn]-Trp	OH	Lactam bridge D1-Orn8	83,36	1345,17	1327,58 *
12749	pGua	[Asp-Met-Leu(5F3)-Arg-Val-Arg-Pro-Orn]-Trp	OH	Lactam bridge D1-Orn8	92,82	1383,18	1383,57
12750	pGua	[Asp-Met-Nle-Arg-Val-Arg-Pro-Orn]-Trp	OH	Lactam bridge D1-Orn8	93,51	1330,83	1329,6
12751	pGua	[Asp-Met-ButGly-Arg-Val-Arg-Pro-Orn]-Trp	OH	Lactam bridge D1-Orn8	92,18	1330,83	1329,6
12752	pGua	[Asp-Met-Leu-Arg-Val-Arg-Aib-Orn]-Trp	OH	Lactam bridge D1-Orn8	90,36	1317,9	1317,58
13628	pGua	[Asp-Met-Leu(4OH)-Arg-Val-Arg-Pro-Orn]-Trp	OH	Lactam bridge D1-Orn8	84,42	1345,17	1345,59
13661	pGua	[Glu-MeCys-Leu-Arg-Val-Arg-Pro-Orn]-Trp	OH	Lactam bridge E1-Orn8	95,49	1328,96	1329,6
13662	pGua	[Glu-bhMet-Leu-Arg-Val-Arg-Pro-Orn]-Trp	OH	Lactam bridge E1-Orn8	96,07	1357,12	1357,65
13663	pGua	[Glu-Eth-Leu-Arg-Val-Arg-Pro-Orn]-Trp	OH	Lactam bridge E1-Orn8	95,96	1357,54	1357,65
13664	pGua	[Glu-Buth-Leu-Arg-Val-Arg-Pro-Orn]-Trp	OH	Lactam bridge E1-Orn8	94,55	1385,16	1385,7
13665	pGua	[Glu-SeMet-Leu-Arg-Val-Arg-Pro-Orn]-Trp	OH	Lactam bridge E1-Orn8	93,19	1390,66	1390,52
13666	pGua	[Glu-Met-3FLeu-Arg-Val-Arg-Pro-Orn]-Trp	OH	Lactam bridge E1-Orn8	90,68	1397,09	1397,59
13667	pGua	[Glu-Met-Nle-Arg-Val-Arg-Pro-Orn]-Trp	OH	Lactam bridge E1-Orn8	96,19	1343,11	1343,62
13668	pGua	[Glu-Met-ButGly-Arg-Val-Arg-Pro-Orn]-Trp	OH	Lactam bridge E1-Orn8	95,76	1343,06	1343,62

13669	pGua	Glu-Met-Leu-Arg-Val-Arg-Pro-Orn-Trp	OH	Linear	95,26	1361,52	1361,64
13670	pGua	Ala-Met-Leu-Arg-Val-Arg-Pro-Ala-Trp	OH	Linear	95,38	1260,16	1260,53
13671	pGua	[Pra-Eth-Leu-Arg-Val-Arg-Pro-Aha]-Trp	OH	CLICK bridge	90,24	1353,21	1353,62
13672	pGua	[Glu-Met-Leu-Arg-Val-Arg-Pro-Orn]-NMeTrp	OH	Lactam bridge E1-Orn8	97,02	1357,11	1357,65
13673	pGua	[Glu-Met-Leu-Arg-Val-Arg-Pro-Orn]-Bta	OH	Lactam bridge E1-Orn8	95,22	1361,06	1360,67
13674	pGua	[Glu-Met-Leu-Arg(NO2)-Val-Arg-Pro-Orn]-Trp	OH	Lactam bridge E1-Orn8	95,01	1388,13	1388,62
13675	pGua	[Glu-Met-Leu-Arg(Me)-Val-Arg-Pro-Orn]-Trp	OH	Lactam bridge E1-Orn8	90,71	1356,98	1357,65
13676	pGua	[Glu-Met-Leu-Cit-Val-Arg-Pro-Orn]-Trp	OH	Lactam bridge E1-Orn8	95,84	1344,21	1344,61
13677	pGua	[Glu-Met-Leu-hArg-Val-Arg-Pro-Orn]-Trp	OH	Lactam bridge E1-Orn8	95,02	1357,17	1357,65
13678	pGua	[Glu-Met-Leu-Cav-Val-Arg-Pro-Orn]-Trp	OH	Lactam bridge E1-Orn8	90,77	1345,68	1345,55
13679	pGua	[Glu-Met-Leu-Arg-Val-Arg(NO2)-Pro-Orn]-Trp	OH	Lactam bridge E1-Orn8	96,39	1388,12	1388,62
13680	pGua	[Glu-Met-Leu-Arg-Val-Arg(Me)-Pro-Orn]-Trp	OH	Lactam bridge E1-Orn8	91,06	1357,5	1357,65
13681	pGua	[Glu-Met-Leu-Arg-Val-Cit-Pro-Orn]-Trp	OH	Lactam bridge E1-Orn8	95,09	1344,2	1344,61
13682	pGua	[Glu-Met-Leu-Arg-Val-hArg-Pro-Orn]-Trp	OH	Lactam bridge E1-Orn8	95,45	1357,33	1357,65
13683	pGua	[Glu-Met-Leu-Arg-Val-Cav-Pro-Orn]-Trp	OH	Lactam bridge E1-Orn8	95,6	1344,88	1345,55
13684	pGua	[Glu-Met-Leu-Arg-Val-Arg-Pro(4NH2)-Orn]-Trp	OH	Lactam bridge E1-Orn8	95,6	1358,24	1358,64
13685	pGua	[Glu-Met-Leu-Arg-Val-Arg-dmPro-Orn]-Trp	OH	Lactam bridge E1-Orn8	95	1370,86	1371,68
13686	pGua	[Glu-Met-Leu-Arg-Val-Arg-Pro(4CF3)-Orn]-Trp	OH	Lactam bridge E1-Orn8	94,09	1411,15	1411,62
13687	pGua	[Glu-Met-Leu-Arg-Val-Arg-Pro(4Ph)-Orn]-Trp	OH	Lactam bridge E1-Orn8	95,38	1419,4	1419,72
13688	pGua	[Glu-Met-Leu-Arg-Val-Arg-Pro(4NH2)]-Ala-Trp	OH	Lactam bridge E1-P7	93,93	1315,04	1315,57
13689	pGua	[Glu-Met-Leu-Arg-Val-Arg-Pro-Orn]-Trt		Lactam bridge E1-Orn7	95,97	1299,92	1299,61
13695	pGua	[Glu-Met-Leu-Arg-Val-Tyr-Arg-Pro-Orn]-Trp	OH	Lactam bridge E1-Orn9	93,09	1506,49	1506,8
14488	pGua	[Glu-Met-Leu-Gly-Arg-Val-Arg-Pro-Orn]-Trp	OH	Lactam bridge E1-Orn9	93,86	1400,68	1400,67
14489	pGua	Glu-Met-Leu-Arg-Val-Cav-Pro-Orn-Trp	OH	Linear	94,63	1363,93	1363,61
14509	pGua	[Glu-SeMet-Leu(5F3)-Arg-Val-Arg-Pro-Orn]-Trp	OH	Lactam bridge E1-Orn8	90,73	1445,14	1444,49
14510	pGua	[Glu-Met-Leu(5F3)-Arg(Me)-Val-Arg-Pro-Orn]-Trp	OH	Lactam bridge E1-Orn8	95,36	1412,12	1411,62
14511	pGua	[Glu-Met-Leu-Arg(Me)-Val-Arg(Me)-Pro-Orn]-Trp	OH	Lactam bridge E1-Orn8	93,76	1371,65	1371,68
14512	pGua	[Glu-Met-Leu-Arg-Val-Arg(Me)-dmPro-Orn]-Trp	OH	Lactam bridge E1-Orn8	91,45	1386,16	1385,7
14514	pGua	[Glu-Met-Leu-Arg-Tyr-Arg-Pro-Orn]-Trp	OH	Lactam bridge E1-Orn8	92,95	1407,95	1407,67

14515	Aaba	[Glu-Met-Leu-Arg-Val-Arg-Pro-Orn]-Trp	OH	Lactam bridge E1-Orn8	91,05	1344,81	1344,61
14516	pGua	[hPra-Met-Leu-Arg-Val-Arg-Pro-Aha]-Trp	OH	CLICK bridge	96,8	1353,6	1353,62
14517	pGua	[Aha-Met-Leu-Arg-Val-Arg-Pro-hPra]-Trp	OH	CLICK bridge	95,22	1353,6	1353,62
14518	pGua	[Pra-Met-Leu-Arg-Val-Arg-Pro-Nva(N3)]-Trp	OH	CLICK bridge	95,46	1353,87	1353,62
14519	pGua	[Nva(N3)-Met-Leu-Arg-Val-Arg-Pro-Pra]-Trp	OH	CLICK bridge	90,11	1353,5	1353,62
14522	pGua	Glu-Met-Leu-Arg	OH	Linear	97,94	709,14	708,84
14523	H	Val-Arg-Pro-Orn-Trp	OH	Linear	98,18	670,81	670,82
15287	pGua	[Glu-Met-Leu-Arg-Val-Arg-Aib-Orn]-Trp	OH	Lactam bridge E1-Orn8	96,38	1332,13	1331,61
15288	pGua	[Glu-Met-Leu-Arg-Val-Tyr-Arg-Pro(4NH2)-Orn]-Trp	OH	Lactam bridge E1-Orn8	94,92	1522,17	1521,8
15290	pGua	[Aha-Met-Leu-Arg-bhVal-Arg-Pro-Pra]-Trp	OH	CLICK bridge	93	1353,3	1353,62
15291	pGua	[Pra-Eth-Leu-Arg-bhVal-Arg-Pro-Aha]-Trp	OH	CLICK bridge	91,81	1367,6	1367,65
15292	pGua	[Glu-Met-Leu-Arg-Val-Tyr-Arg-Pro-Orn]-Trp	OH	Lactam bridge E1-Orn8	92,01	1507,13	1506,8
15293	H	dArg-[Glu-Met-Leu-Arg-Val-Arg-Pro-Orn]-Trp	OH	Lactam bridge E2-Orn9	94,01	1338,99	1338,65
15363	pGua	[Aha-Met-Leu-Arg-bhVal-Arg-Pro-Pra]-Bta	OH	CLICK bridge	96,19	1371,2	1370,67
15364	pGua	[Aha-Met-Leu-Arg-bhVal-Arg-Pro(4NH2)-Pra]-Trp	OH	CLICK bridge	97,03	1368,65	1368,64
15365	pGua	[Aha-SeMet-Leu-Arg-bhVal-Arg-Pro-Pra]-Trp	OH	CLICK bridge	95,06	1401,47	1400,65
15366	pGua	[Aha-Met-Leu-Arg-bhVal-Arg-Pro-Pra]	tryptamine	CLICK bridge	92,56	1309,64	1309,61
15367	pGua	[Aha-NMeMet-Leu-Arg-bhVal-Arg-Pro-Pra]-Trp	OH	CLICK bridge	94,08	1368,11	1367,65
15368	pGua	[Aha-Met-NMeLeu-Arg-bhVal-Arg-Pro-Pra]-Trp	OH	CLICK bridge	93,12	1368,38	1367,65
15369	pGua	[Aha-Met-Leu-NMeArg-bhVal-Arg-Pro-Pra]-Trp	OH	CLICK bridge	92	1367,51	1367,65
15370	pGua	[Aha-Met-Leu-Arg-bhVal-NMeArg-Pro-Pra]-Trp	OH	CLICK bridge	90,62	1367,81	1367,65
18169	pGua	[Aha-Nle-Leu-Arg-bhVal-Arg-Pro-Pra]-Trp	OH	CLICK bridge	97,18	1335,1	1335,59

The abbreviations used are as follows (in alphabetical order): 2Abu=2-aminobutyric acid; 3FLeu=5,5,5-trifluoro-DL-leucine; Aaba= 4-((aminocarbonyl) amino) benzoic acid; Aha=L-azidohomoalanine; Aib= α-aminoisobutyric acid; Arg(NO2)=Nω-nitro-L-arginine; ArgBzi=Arg benzimidazole; Arg(Me)=Nω,ω-dimethyl-L-arginine (symmetrical); Ava=L-5-aminovaleric acid, L-5-aminopentanoic acid; bhMet=L-β-homomethionine; bhVal=L-β-homovaline; Bta=L-3-benzothienylalanine; ButGly=L-α-t-butylglycine ; Buth=buthionine; Cav=Canavanine; Cit=L-citrulline; Dab=L-2,4-diamino butyric acid; Dap=L-2,3-diamino propionic acid; dhLeu=4,5-dehydro-L-Leucine ; dmPro=5,5-dimethyl-L-proline; Eth=DL-ethionine; Eth=L-ethionine; hArg= homoArginine ; hPra=homo-propargylglycine; Leu(4OH)=4-hydroxy-L-Leucine; Leu(5F3)=5,5,5-trifluoro-DL-leucine ; MeCys=S-methyl-L-cysteine; NMeArg=N-methyl-L-arginine; NMeLeu=N-methyl-L-leucine; NMeMet=N-methyl-L-methionine; NMeTrp=N-methyl-Trp; Nva(N3)=5-azido-L-norvaline; pGua= p-guanidino benzoic acid; Pra=propargylglycine; Pro(4CF3)=4-trifluoromethyl-L-proline; Pro(4NH2)=4(S)-amino-L-proline; Pro(4Ph)=4(S)-phenyl-L-proline; SeMet=L-seleno-methionine; Trt=tryptamine.

Purity was obtaiend by liquid chrmoatography analyses, eithet uPLC or HPLC coupled with a ???mass spectrometer ().

Table S2. **Blood biochemistry of diet-induced obese mice with and without treatment by the MCHR1 antagonist GPS18169. Individual data**

Gr.	Treatment	Route	Dose	No.	Insulin (pg/mL)			Total Cholesterol (mg/dL)			Triglyceride (mg/dL)		
					Day 56	Day 113	Day 141	Day 56	Day 113	Day 141	Day 56	Day 113	Day 141
1	Vehicle (Normal Diet)	IP	10 mL/kg QD x 12 wks	1	403.1	801.8	1027.0	86.8	90.3	96.6	66.0	81.4	72.2
				2	256.6	587.5	817.7	106.4	106.8	99.2	66.0	98.0	67.4
				3	628.6	483.9	913.3	104.4	111.6	109.1	81.4	109.7	89.4
				4	288.9	204.3	782.2	93.8	99.8	111.3	47.7	75.8	110.2
				5	630.5	405.0	552.5	90.5	91.3	68.7	85.4	66.8	63.2
				6	485.2	298.1	1234.7	92.5	118.2	115.1	54.8	114.7	112.2
				7	261.9	409.5	595.0	97.9	104.8	101.5	73.1	93.0	74.0
				8	243.3	480.1	362.5	110.3	109.9	111.3	68.3	75.7	72.5
				9	442.7	274.4	655.9	97.3	101.4	88.4	55.2	70.2	46.7
				10	170.3	710.3	665.8	101.5	97.0	111.0	97.3	78.0	75.7
				Mean	381.1	438.3	760.7	98.1	103.1	101.2	69.5	86.3	78.3
				SEM	51.7	57.2	79.7	2.4	2.8	4.5	4.8	5.3	6.4
2	Vehicle (High-fat Diet)	IP	10 mL/kg QD x 12 wks	1	575.0	1046.3	540.6	131.2	115.1	115.5	148.0	145.2	159.8
				2	1837.2	3540.2	4068.9	170.1	189.0	246.4	119.8	115.0	185.9
				3	613.7	2283.2	911.4	152.6	154.4	121.4	159.2	148.8	117.2
				4	597.5	4421.8	2041.5	196.7	183.9	139.7	129.9	124.8	110.6
				5	503.5	1104.1	300.1	124.4	141.6	110.8	136.7	138.6	80.0
				6	1048.1	2154.5	2710.4	155.0	175.0	196.6	88.4	91.9	126.0
				7	1125.7	3562.6	1710.3	173.4	191.9	138.0	117.2	116.7	105.2
				8	1046.9	1489.5	1187.4	167.5	174.1	145.7	118.1	124.1	91.5
				9	720.8	3284.4	4949.3	180.3	186.5	255.1	115.4	112.0	170.6
				10	801.8	2712.7	1746.3	104.6	124.5	107.4	100.6	103.6	88.4
				Mean	887.0	2543.0†	2016.6†	155.6†	163.6†	157.7†	123.4†	122.1†	123.5†
				SEM	126.9	383.3	477.2	8.9	8.9	17.5	6.7	5.7	11.6
3	PT#1223046 (SV-989) GPS18169-002	IP	10 mg/kg QD x 12 wks	1	927.2	2534.7	1421.2	161.8	156.9	143.8	129.6	87.6	94.5
				2	1329.6	798.1	173.1	158.2	105.0	122.6	125.9	100.4	92.7
				3	1182.6	230.6	160.0	164.4	101.8	127.5	130.4	114.3	86.5
				4	1450.9	3654.4	884.1	196.8	160.4	161.2	128.8	85.0	112.3
				5	809.7	1653.6	912.6	181.9	141.3	164.8	108.3	105.3	114.3
				6	2028.0	2230.3	714.0	131.2	166.7	136.5	113.9	99.6	112.3
				7	663.3	401.8	265.8	156.6	127.1	125.5	136.0	113.6	90.4
				8	834.1	1108.3	4093.9	118.6	106.8	105.0	104.2	144.0	104.9
				9	388.3	2596.4	718.9	153.4	181.3	132.5	118.4	135.1	102.7
				10	1066.2	288.9	226.6	133.0	110.3	102.1	133.9	98.5	110.4
				Mean	1068.0	1549.7	957.0*	155.6	135.7	132.1	122.9	108.3	102.1
				SEM	145.9	370.8	371.8	7.4	9.3	6.6	3.5	6.0	3.3
4	PT#1223046 (SV-989) GPS18169-002	IP	5 mg/kg QD x 12 wks	1	1229.3	526.2	399.2	146.1	103.0	91.1	140.8	101.4	95.2
				2	1153.3	382.5	438.2	152.1	123.9	97.4	103.6	97.9	53.7
				3	637.9	3588.7	834.1	176.9	155.4	126.0	129.4	114.2	128.4
				4	768.1	207.0	192.8	144.8	102.8	103.2	137.4	106.0	90.4
				5	720.8	5018.2	2296.7	203.0	221.6	242.4	110.7	106.6	131.6
				6	370.9	875.6	820.1	179.4	112.1	128.3	98.7	105.4	98.0
				7	1716.8	3494.0	1346.9	155.9	191.5	187.5	134.0	115.8	136.5
				8	961.7	1027.0	494.7	132.6	159.3	133.6	126.7	143.9	141.7
				9	1037.2	2735.1	528.0	159.3	172.7	129.4	114.9	155.2	135.1
				10	499.1	314.5	253.2	133.2	88.5	117.2	122.0	106.2	78.6
				Mean	909.5	1983.8	760.4*	158.3	143.1	135.6	121.8	115.3	108.9
				SEM	125.6	554.7	201.4	7.0	13.9	14.6	4.6	6.0	9.4

Gr.	Treatment	Route	Dose	No.	AST (U/L)			ALT (U/L)			Uric Acid (mg/dL)		
					Day 56	Day 113	Day 141	Day 56	Day 113	Day 141	Day 56	Day 113	Day 141
1	Vehicle (Normal Diet)	IP	10 mL/kg QD x 12 wks	1	121.5	79.2	68.1	41.1	51.2	47.4	0.9	1.2	1.2
				2	114.3	225.2	91.2	96.0	56.0	120.3	0.9	1.2	1.2
				3	104.4	129.6	158.4	30.9	42.0	61.2	0.9	1.6	0.9
				4	124.5	155.2	81.9	45.0	55.6	42.9	1.2	1.2	1.2
				5	147.3	243.6	289.2	44.4	94.4	75.9	1.5	2.0	1.5
				6	86.4	232.0	88.8	30.9	73.6	73.8	1.2	1.6	1.2
				7	161.7	100.0	156.6	62.7	58.4	93.9	1.2	1.2	1.2
				8	162.6	236.8	182.7	36.6	119.6	55.8	0.9	1.2	1.2
				9	208.5	71.6	167.4	53.1	51.2	110.4	0.9	1.2	0.9
				10	149.4	82.8	101.7	51.6	50.8	54.3	0.9	0.8	1.2
				Mean	138.1	155.6	138.6	49.2	65.3	73.6	1.1	1.3	1.2
				SEM	11.1	22.8	21.2	6.1	7.6	8.4	0.1	0.1	0.1
2	Vehicle (High-fat Diet)	IP	10 mL/kg QD x 12 wks	1	95.1	113.6	145.8	26.7	64.4	93.6	1.8	1.2	1.2
				2	189.3	219.6	214.2	99.3	170.4	134.1	1.2	1.2	1.5
				3	125.4	138.0	114.9	55.5	76.4	102.3	1.5	1.6	0.9
				4	124.5	164.4	198.9	89.7	111.2	132.9	1.2	1.6	1.2
				5	130.8	177.6	115.8	39.3	77.6	55.2	1.2	1.2	1.2
				6	120.0	132.0	217.5	54.6	80.4	118.5	0.9	1.2	1.5
				7	123.3	144.0	224.7	48.0	73.2	94.5	1.5	1.6	1.2
				8	116.1	139.6	188.1	49.8	73.6	84.0	1.2	1.2	1.5
				9	231.9	203.2	217.2	96.6	123.6	99.0	1.2	1.6	1.2
				10	174.9	199.2	122.1	66.0	80.8	51.9	0.9	1.2	1.2
				Mean	143.1	163.1	175.9	62.6	93.2	96.6	1.3	1.4	1.3
				SEM	13.2	11.2	14.6	7.9	10.4	8.9	0.1	0.1	0.1
3	PT#1223046 (SV-989) GPS18169-002	IP	10 mg/kg QD x 12 wks	1	179.7	171.2	172.3	62.4	105.6	109.2	1.2	1.2	1.2
				2	132.0	128.0	178.5	68.7	58.4	85.8	1.2	0.8	1.2
				3	126.9	84.8	153.1	68.1	60.4	135.9	1.5	1.2	1.2
				4	118.8	108.4	196.8	85.5	104.8	117.0	1.5	1.6	0.9
				5	210.0	193.6	170.1	67.2	110.4	76.2	1.2	1.2	1.2
				6	115.5	136.8	175.2	49.8	74.4	126.9	0.9	1.2	1.2
				7	150.6	123.2	153.4	57.6	85.2	65.7	1.2	0.8	1.5
				8	179.1	354.8	258.7	61.5	85.6	140.1	1.5	1.2	1.2
				9	154.2	249.6	156.1	48.9	176.4	54.0	1.2	1.2	1.2
				10	117.0	168.4	198.7	52.5	40.8	90.6	1.5	1.2	1.5
				Mean	148.4	171.9	181.3	62.2	90.2	100.1	1.3	1.2	1.2
				SEM	10.2	25.2	10.0	3.5	12.0	9.5	0.1	0.1	0.1
4	PT#1223046 (SV-989) GPS18169-002	IP	5 mg/kg QD x 12 wks	1	103.5	148.8	195.9	37.2	49.6	46.5	1.8	1.6	1.2
				2	154.8	116.8	210.9	43.5	36.0	132.6	1.5	1.2	1.2
				3	198.6	219.6	152.7	85.8	186.4	70.2	1.2	1.2	1.2
				4	112.5	102.4	231.0	45.0	36.8	83.4	1.2	0.8	1.2
				5	172.2	154.8	237.9	80.7	162.4	135.9	1.2	1.2	1.5
				6	165.9	102.0	137.1	94.8	58.0	48.6	1.2	1.2	1.2
				7	147.9	127.6	150.0	43.5	73.2	80.7	0.9	0.8	1.5
				8	111.3	167.2	135.6	46.2	141.6	116.7	1.2	1.2	1.2
				9	160.2	186.4	119.7	93.6	94.4	47.7	1.2	1.2	0.9
				10	123.6	154.0	158.1	39.6	67.2	103.8	1.2	1.2	1.2
				Mean	145.1	148.0	172.9	61.0	90.6	86.6	1.3	1.2	1.2
				SEM	9.9	11.8	13.4	7.7	17.1	10.9	0.1	0.1	0.1

Gr.	Treatment	Route	Dose	No.	Creatinine (mg/dL)			Potassium (µEq/mL)			Sodium (µEq/mL)		
					Day 56	Day 113	Day 141	Day 56	Day 113	Day 141	Day 56	Day 113	Day 141
1	Vehicle (Normal Diet)	IP	10 mL/kg QD x 12 wks	1	0.24	0.16	0.30	5.01	5.24	5.16	154.59	141.84	138.84
				2	0.39	0.36	0.36	6.42	5.72	5.40	162.78	150.84	133.62
				3	0.27	0.28	0.42	5.25	5.24	6.12	154.68	134.20	139.80
				4	0.27	0.24	0.33	5.25	5.84	6.00	155.43	148.72	136.20
				5	0.42	0.32	0.39	6.15	6.00	5.61	156.60	146.04	129.12
				6	0.27	0.28	0.33	5.67	5.68	5.67	155.82	151.24	129.42
				7	0.27	0.24	0.24	5.91	5.84	5.67	160.02	149.20	140.97
				8	0.39	0.36	0.36	5.85	5.68	6.15	157.62	158.28	137.85
				9	0.33	0.24	0.36	5.94	5.28	5.58	155.85	141.36	136.56
				10	0.24	0.24	0.30	5.64	5.68	5.94	155.94	148.04	132.63
				Mean	0.31	0.27	0.34	5.71	5.62	5.73	156.93	146.98	135.50
				SEM	0.02	0.02	0.02	0.14	0.09	0.10	0.82	2.09	1.32
2	Vehicle (High-fat Diet)	IP	10 mL/kg QD x 12 wks	1	0.24	0.28	0.33	6.24	5.52	5.73	154.44	158.56	140.25
				2	0.27	0.28	0.33	6.09	6.20	6.12	156.03	169.68	149.07
				3	0.30	0.28	0.36	6.21	5.88	6.24	157.68	147.28	139.44
				4	0.39	0.24	0.39	5.67	5.44	7.02	155.85	138.40	139.26
				5	0.24	0.32	0.27	5.73	6.72	5.55	150.18	154.48	137.76
				6	0.30	0.40	0.42	6.66	5.12	6.18	153.93	135.96	133.56
				7	0.30	0.20	0.33	6.15	5.40	6.09	159.75	155.72	134.67
				8	0.30	0.28	0.30	6.48	6.00	5.52	155.94	143.24	137.55
				9	0.27	0.36	0.45	6.09	5.44	5.64	151.83	151.24	132.27
				10	0.42	0.24	0.33	6.39	5.28	5.31	157.38	144.52	135.36
				Mean	0.30	0.29	0.35	6.17	5.70	5.94	155.30	149.91	137.92
				SEM	0.02	0.02	0.02	0.10	0.16	0.16	0.89	3.21	1.50
3	PT#1223046 (SV-989) GPS18169-002	IP	10 mg/kg QD x 12 wks	1	0.27	0.28	0.24	6.39	5.76	5.40	155.85	149.24	127.08
				2	0.36	0.24	0.27	6.21	5.80	5.37	154.29	147.48	138.99
				3	0.27	0.24	0.33	5.79	5.40	4.89	150.54	159.28	139.53
				4	0.39	0.24	0.33	6.60	5.04	5.58	159.75	123.08	141.12
				5	0.33	0.24	0.39	6.30	5.48	6.30	156.09	145.84	136.47
				6	0.27	0.20	0.27	5.64	6.44	5.64	157.62	143.88	125.52
				7	0.36	0.36	0.39	6.36	5.64	5.01	155.94	143.32	140.70
				8	0.30	0.20	0.33	5.91	5.36	6.18	154.62	145.20	138.57
				9	0.27	0.36	0.30	6.00	7.16	5.40	154.80	171.16	135.33
				10	0.30	0.32	0.33	5.97	5.40	5.43	157.32	152.44	139.35
				Mean	0.31	0.27	0.32	6.12	5.75	5.52	155.68	148.09	136.27
				SEM	0.01	0.02	0.02	0.10	0.20	0.14	0.77	3.88	1.75
4	PT#1223046 (SV-989) GPS18169-002	IP	5 mg/kg QD x 12 wks	1	0.30	0.24	0.27	6.12	5.72	5.22	153.00	148.24	142.77
				2	0.36	0.24	0.30	6.27	6.32	5.61	155.70	149.64	139.44
				3	0.33	0.32	0.39	6.99	6.60	5.94	154.47	152.32	151.35
				4	0.24	0.28	0.27	6.03	5.52	5.76	151.32	144.28	145.62
				5	0.27	0.24	0.36	6.63	6.24	6.24	161.10	153.16	137.22
				6	0.42	0.40	0.33	6.18	6.40	6.12	160.56	148.44	136.95
				7	0.33	0.32	0.39	5.55	6.04	5.22	164.22	146.64	131.19
				8	0.30	0.20	0.33	6.51	6.32	6.06	155.04	150.48	136.26
				9	0.30	0.32	0.33	5.64	6.76	5.10	156.09	151.72	141.48
				10	0.33	0.32	0.36	6.21	5.80	5.31	157.05	140.32	142.95
				Mean	0.32	0.29	0.33	6.21	6.17	5.66	156.86	148.52	140.52
				SEM	0.02	0.02	0.01	0.14	0.13	0.13	1.26	1.25	1.78

Table S3: Comparison of the adipose tissue weights between lean, high fat diet fed and GPS18169-treated mice. **Individual data.**

Gr.	Treatment	Route	Dose	No.	B.W. (g) Day 141	Adipose Tissue Weight (g)				
						Epididymal	Mesenteric	Retroperitoneal	Inguinal	Brown fat
1	Vehicle (Normal Diet)	IP	10 mL/kg QD x 12 wks	1	26	0.695	0.328	0.183	0.320	0.091
				2	26	0.657	0.288	0.184	0.242	0.082
				3	29	1.011	0.423	0.338	0.596	0.111
				4	28	0.823	0.262	0.229	0.439	0.113
				5	29	0.593	0.388	0.166	0.411	0.143
				6	29	1.058	0.436	0.378	0.561	0.103
				7	27	0.527	0.298	0.158	0.334	0.072
				8	26	0.740	0.384	0.190	0.395	0.093
				9	25	0.438	0.261	0.138	0.334	0.088
				10	29	0.805	0.384	0.230	0.385	0.089
				Mean	27.2	0.735	0.345	0.219	0.402	0.099
				SEM	0.5	0.063	0.021	0.025	0.034	0.006
2	Vehicle (High-fat Diet)	IP	10 mL/kg QD x 12 wks	1	30	1.282	0.320	0.360	1.018	0.128
				2	48	1.889	1.051	0.853	2.775	0.241
				3	33	2.147	0.472	0.520	1.299	0.113
				4	39	1.634	0.564	0.708	1.953	0.161
				5	27	1.362	0.508	0.404	0.587	0.068
				6	51	2.844	0.877	1.009	2.177	0.232
				7	40	2.152	0.587	0.780	1.714	0.177
				8	41	2.140	0.782	0.831	1.715	0.159
				9	51	1.828	1.112	1.643	2.693	0.225
				10	40	2.446	0.609	0.681	1.316	0.132
				Mean	40.0†	1.972†	0.688†	0.779†	1.725†	0.164†
				SEM	2.6	0.152	0.082	0.116	0.222	0.018
3	PT#1223046 (SV-989) GPS18169-002	IP	10 mg/kg QD x 12 wks	1	39	2.597	0.596	0.657	2.002	0.135
				2	26	1.575	0.206	0.178	0.214	0.056
				3	26	1.546	0.262	0.091	0.248	0.047
				4	33	1.643	0.527	0.433	1.558	0.103
				5	32	1.969	0.322	0.515	1.502	0.105
				6	33	1.962	0.419	0.468	1.223	0.087
				7	25	2.473	0.127	0.095	0.399	0.058
				8	29	2.416	0.318	0.374	0.711	0.101
				9	31	2.356	0.360	0.294	0.891	0.097
				10	25	1.373	0.203	0.094	0.175	0.052
				Mean	29.9*	1.991	0.334*	0.320*	0.892*	0.084*
				SEM	1.4	0.141	0.047	0.064	0.206	0.009
4	PT#1223046 (SV-989) GPS18169-002	IP	5 mg/kg QD x 12 wks	1	24	2.468	0.206	0.111	0.333	0.062
				2	26	2.159	0.238	0.076	0.131	0.050
				3	31	1.403	0.243	0.500	1.565	0.095
				4	23	1.914	0.077	0.043	0.109	0.043
				5	47	1.806	1.056	1.008	3.016	0.282
				6	25	1.288	0.362	0.151	0.338	0.060
				7	36	2.656	0.676	0.501	2.115	0.136
				8	28	1.535	0.190	0.333	0.572	0.105
				9	28	1.467	0.328	0.358	0.882	0.086
				10	26	2.128	0.137	0.132	0.160	0.041
				Mean	29.8*	1.882	0.351*	0.321*	0.922*	0.096*
				SEM	2.4	0.148	0.094	0.093	0.314	0.023

Gr.	Treatment	Route	Dose	No.	B.W. (g) Day 141	Adipose Tissue Weight per 100g B.W. Ratio (%)				
						Epididymal	Mesenteric	Retroperitoneal	Inguinal	Brown fat
1	Vehicle (Normal Diet)	IP	10 mL/kg QD x 12 wks	1	26	2.67	1.26	0.70	1.23	0.35
				2	26	2.53	1.11	0.71	0.93	0.32
				3	29	3.49	1.46	1.17	2.06	0.38
				4	28	2.94	0.94	0.82	1.57	0.40
				5	29	2.04	1.34	0.57	1.42	0.49
				6	29	3.65	1.50	1.30	1.93	0.36
				7	27	1.95	1.10	0.59	1.24	0.27
				8	26	2.85	1.48	0.73	1.52	0.36
				9	25	1.75	1.04	0.55	1.34	0.35
				10	29	2.78	1.32	0.79	1.33	0.31
				Mean	27.2	2.66	1.26	0.79	1.46	0.36
				SEM	0.5	0.20	0.06	0.08	0.11	0.02
2	Vehicle (High-fat Diet)	IP	10 mL/kg QD x 12 wks	1	30	4.27	1.07	1.20	3.39	0.43
				2	48	3.94	2.19	1.78	5.78	0.50
				3	33	6.51	1.43	1.58	3.94	0.34
				4	39	4.19	1.45	1.82	5.01	0.41
				5	27	5.04	1.88	1.50	2.17	0.25
				6	51	5.58	1.72	1.98	4.27	0.45
				7	40	5.38	1.47	1.95	4.29	0.44
				8	41	5.22	1.91	2.03	4.18	0.39
				9	51	3.58	2.18	3.22	5.28	0.44
				10	40	6.12	1.52	1.70	3.29	0.33
				Mean	40.0†	4.98†	1.68	1.87†	4.16†	0.40
				SEM	2.6	0.30	0.11	0.17	0.33	0.02
3	PT#1223046 (SV-989) GPS18169-002	IP	10 mg/kg QD x 12 wks	1	39	6.66	1.53	1.68	5.13	0.35
				2	26	6.06	0.79	0.68	0.82	0.22
				3	26	5.95	1.01	0.35	0.95	0.18
				4	33	4.98	1.60	1.31	4.72	0.31
				5	32	6.15	1.01	1.61	4.69	0.33
				6	33	5.95	1.27	1.42	3.71	0.26
				7	25	9.89	0.51	0.38	1.60	0.23
				8	29	8.33	1.10	1.29	2.45	0.35
				9	31	7.60	1.16	0.95	2.87	0.31
				10	25	5.49	0.81	0.38	0.70	0.21
				Mean	29.9*	6.71	1.08*	1.01*	2.77	0.27*
				SEM	1.4	0.47	0.11	0.17	0.55	0.02
4	PT#1223046 (SV-989) GPS18169-002	IP	5 mg/kg QD x 12 wks	1	24	10.28	0.86	0.46	1.39	0.26
				2	26	8.30	0.92	0.29	0.50	0.19
				3	31	4.53	0.78	1.61	5.05	0.31
				4	23	8.32	0.33	0.19	0.47	0.19
				5	47	3.84	2.25	2.14	6.42	0.60
				6	25	5.15	1.45	0.60	1.35	0.24
				7	36	7.38	1.88	1.39	5.88	0.38
				8	28	5.48	0.68	1.19	2.04	0.38
				9	28	5.24	1.17	1.28	3.15	0.31
				10	26	8.18	0.53	0.51	0.62	0.16
				Mean	29.8*	6.67	1.08*	0.97*	2.69	0.30*
				SEM	2.4	0.66	0.19	0.21	0.73	0.04

Table S4: Food intake in high-fat diet-induced obesity model in C57BL/6 mice during treatment with GPS18169. Individual data.

Gr.	Treatment	Route	Dose	No.	Food Intake (g/day) Day 57	Day 61	Day 64	Day 68	Day 71	Day 75	Day 78	Day 82
1	Vehicle (Normal Diet)	IP	10 mL/kg QD x 12 wks	1	3.8	3.7	3.0	4.7	3.5	4.3	3.8	4.0
				2	4.8	5.0	4.3	4.7	3.5	4.7	5.0	4.3
				3	3.8	4.0	3.8	4.3	3.5	4.0	4.0	4.3
				4	4.0	4.0	3.5	4.3	3.5	4.3	3.8	3.7
				5	4.8	4.7	4.3	5.0	3.5	5.3	4.3	4.7
				6	4.3	3.0	3.8	4.7	3.8	4.0	4.3	4.0
				7	4.3	3.0	4.0	4.7	3.3	4.3	4.0	4.0
				8	4.3	3.7	4.0	4.3	3.3	3.7	4.0	3.7
				9	4.3	3.7	3.5	4.3	3.3	3.7	4.0	4.0
				10	4.3	3.7	4.3	5.3	3.5	3.3	3.8	4.0
				Mean	4.2	3.8	3.9	4.6	3.5	4.2	4.1	4.1
				SEM	0.1	0.2	0.1	0.1	0.0	0.2	0.1	0.1
2	Vehicle (High-fat Diet)	IP	10 mL/kg QD x 12 wks	1	3.0	3.3	3.5	3.3	3.5	3.7	3.3	3.3
				2	2.8	2.7	3.0	3.0	3.0	3.3	3.3	3.0
				3	2.8	3.0	2.5	3.3	2.8	3.7	3.3	3.3
				4	2.8	2.5	3.0	2.7	2.8	3.3	3.3	3.0
				5	2.5	3.7	2.8	3.0	3.0	3.0	3.0	2.7
				6	3.8	2.7	3.3	3.0	3.3	3.0	3.8	3.7
				7	3.0	3.0	3.0	3.3	3.0	3.0	3.3	3.3
				8	3.0	3.0	3.3	3.0	2.8	3.3	3.5	3.0
				9	2.8	3.0	3.0	3.0	3.0	3.7	3.0	3.0
				10	3.5	3.3	3.3	3.3	3.5	2.7	3.5	3.3
				Mean	3.0†	3.0†	3.1†	3.1†	3.1	3.3†	3.3†	3.2†
				SEM	0.1	0.1	0.1	0.1	0.1	0.1	0.1	0.1
3	PT#1223046 (SV-989) GPS18169-002	IP	10 mg/kg QD x 12 wks	1	3.3	3.7	4.0	3.0	3.0	3.3	3.5	3.7
				2	2.8	3.0	2.8	3.3	2.8	3.0	3.0	3.0
				3	3.0	3.0	3.3	3.0	2.8	3.7	3.0	2.7
				4	3.0	2.7	2.8	3.0	2.5	3.3	3.3	2.7
				5	3.0	3.7	3.0	3.0	3.3	3.0	3.0	3.0
				6	2.8	3.0	3.0	3.0	3.0	3.0	3.0	3.0
				7	2.8	3.3	3.0	3.3	2.8	3.0	2.8	3.0
				8	3.0	3.3	3.3	2.7	2.8	3.3	2.8	3.0
				9	2.8	2.7	3.0	3.0	3.3	3.3	3.0	3.0
				10	3.3	2.7	3.0	2.7	2.5	2.7	2.5	2.7
				Mean	3.0	3.1	3.1	3.0	2.9	3.2	3.0	3.0
				SEM	0.1	0.1	0.1	0.1	0.1	0.1	0.1	0.1
4	PT#1223046 (SV-989) GPS18169-002	IP	5 mg/kg QD x 12 wks	1	3.0	2.7	2.8	2.7	2.8	3.0	2.8	3.0
				2	3.0	3.7	3.5	3.3	3.5	3.3	2.8	2.7
				3	2.8	3.0	2.8	3.0	2.5	3.0	2.5	3.3
				4	2.8	2.7	2.8	3.0	2.5	3.0	3.3	2.7
				5	3.8	3.3	2.8	3.0	3.0	3.3	2.5	3.3
				6	2.8	3.0	3.3	3.0	3.0	3.3	2.8	3.3
				7	3.0	2.7	3.0	2.7	3.0	3.3	3.0	2.7
				8	3.0	3.0	3.5	3.3	2.8	3.0	3.3	2.7
				9	3.0	2.7	3.0	3.0	2.8	3.0	2.8	2.7
				10	3.3	3.0	2.5	3.3	2.8	3.0	2.8	3.3
				Mean	3.0	3.0	3.0	3.0	2.9	3.1	2.9	3.0
				SEM	0.1	0.1	0.1	0.1	0.1	0.0	0.1	0.1

[illegible]

Gr.	Treatment	Route	Dose	No.	Food Intake (g/day)							
					Day 85	Day 89	Day 92	Day 96	Day 99	Day 103	Day 106	Day 110
1	Vehicle (Normal Diet)	IP	10 mL/kg QD x 12 wks	1	3.8	3.7	3.8	3.0	3.8	3.7	3.3	3.4
				2	4.0	4.3	3.5	4.0	3.5	3.0	3.5	3.6
				3	3.3	3.7	3.3	4.3	3.8	3.0	3.3	3.1
				4	3.8	3.7	3.3	4.3	3.3	2.7	3.5	3.4
				5	3.8	4.7	4.0	4.3	4.3	4.3	3.8	3.6
				6	3.8	3.7	3.3	3.3	3.3	2.7	3.3	3.6
				7	3.5	3.3	2.8	3.7	3.8	2.3	2.8	3.3
				8	3.5	3.7	3.0	3.3	3.3	2.7	3.5	3.3
				9	3.0	4.0	3.0	3.7	3.5	3.3	3.0	3.3
				10	3.5	4.3	2.8	4.3	3.5	3.3	2.5	4.0
				Mean	3.6	3.9	3.3	3.8	3.6	3.1	3.2	3.5
				SEM	0.1	0.1	0.1	0.2	0.1	0.2	0.1	0.1
2	Vehicle (High-fat Diet)	IP	10 mL/kg QD x 12 wks	1	2.5	3.0	3.0	3.0	2.8	3.3	3.3	2.7
				2	3.5	3.0	3.8	3.7	3.3	3.3	3.0	3.0
				3	3.0	3.3	3.0	2.7	3.0	3.7	3.3	2.7
				4	3.0	2.7	3.0	3.3	3.0	2.7	2.8	3.1
				5	3.3	2.7	3.8	2.7	2.8	3.7	3.0	2.6
				6	3.0	3.7	3.0	3.0	3.0	2.7	3.0	3.3
				7	3.0	3.0	2.8	2.7	2.8	3.3	2.8	3.3
				8	3.3	3.3	3.3	3.7	3.3	3.3	3.3	2.9
				9	3.0	3.0	3.0	3.3	2.8	3.0	2.8	2.9
				10	3.3	3.3	2.8	2.7	2.8	3.0	3.0	3.1
				Mean	3.1†	3.1†	3.1	3.1†	2.9†	3.2	3.0	3.0†
				SEM	0.1	0.1	0.1	0.1	0.1	0.1	0.1	0.1
3	PT#1223046 (SV-989) GPS18169-002	IP	10 mg/kg QD x 12 wks	1	3.3	3.0	3.0	3.3	3.3	3.0	3.0	3.0
				2	2.8	3.3	2.8	2.7	3.3	3.3	3.3	3.1
				3	3.3	2.7	3.3	3.7	2.8	3.0	3.3	3.0
				4	2.8	2.7	2.8	3.0	2.8	3.7	2.5	2.9
				5	3.3	3.0	3.0	4.0	2.8	3.7	2.8	2.7
				6	3.0	3.0	3.3	3.3	2.5	2.7	3.0	2.7
				7	2.8	3.3	3.0	2.7	2.8	3.0	3.0	2.9
				8	2.8	3.0	2.5	3.0	3.0	2.7	3.0	2.7
				9	3.0	2.7	3.0	3.0	2.8	3.0	2.8	2.7
				10	2.5	3.3	3.0	3.7	2.8	3.7	3.3	3.1
				Mean	2.9	3.0	3.0	3.2	2.9	3.2	3.0	2.9
				SEM	0.1	0.1	0.1	0.1	0.1	0.1	0.1	0.1
4	PT#1223046 (SV-989) GPS18169-002	IP	5 mg/kg QD x 12 wks	1	3.3	3.7	3.3	3.7	3.0	2.7	2.8	3.0
				2	3.0	3.3	2.8	3.0	2.8	3.0	3.0	3.1
				3	2.8	3.7	3.0	3.3	2.5	3.3	2.8	3.1
				4	3.0	3.3	2.8	2.7	3.0	3.7	3.0	3.0
				5	2.8	3.0	3.0	3.0	2.8	2.7	3.3	3.0
				6	3.3	3.3	3.3	2.7	3.3	3.7	2.8	2.7
				7	2.8	2.7	3.3	2.7	3.0	3.7	2.8	2.7
				8	2.5	3.0	2.8	3.3	2.8	3.3	3.0	2.7
				9	3.0	3.0	3.0	3.3	2.8	3.0	2.8	2.6
				10	3.0	2.7	2.8	2.7	2.8	3.7	2.5	2.7
				Mean	2.9	3.2	3.0	3.0	2.9	3.3	2.9	2.9
				SEM	0.1	0.1	0.1	0.1	0.1	0.1	0.1	0.1

[illegible]

Gr.	Treatment	Route	Dose	No.	Food Intake (g/day)						
					Day 117	Day 120	Day 124	Day 127	Day 131	Day 134	Day 138
1	Vehicle (Normal Diet)	IP	10 mL/kg QD x 12 wks	1	3.7	3.0	3.3	3.5	3.7	3.5	4.0
				2	4.0	3.8	4.3	3.5	3.3	2.8	3.5
				3	3.7	3.0	4.0	3.3	3.3	4.3	3.0
				4	3.7	3.5	4.0	3.5	3.7	3.5	4.0
				5	4.0	4.0	4.7	4.0	4.0	3.3	3.5
				6	3.7	3.3	3.7	3.3	4.0	4.3	3.5
				7	3.3	3.0	3.7	3.5	3.7	3.5	3.0
				8	3.7	3.3	3.3	3.3	3.3	3.5	3.5
				9	4.0	3.0	2.7	2.8	3.7	4.0	4.0
				10	4.0	3.8	3.3	4.0	4.0	4.0	3.5
				Mean	3.8	3.4	3.7	3.4	3.7	3.7	3.6
				SEM	0.1	0.1	0.2	0.1	0.1	0.1	0.1
2	Vehicle (High-fat Diet)	IP	10 mL/kg QD x 12 wks	1	3.3	3.8	3.3	2.8	3.3	3.5	3.0
				2	3.3	3.0	3.0	2.5	3.0	3.0	3.5
				3	2.7	2.5	2.7	2.8	3.0	2.8	2.5
				4	3.3	2.8	3.3	4.0	3.3	2.8	3.0
				5	2.7	2.5	2.7	2.5	3.3	3.3	2.5
				6	3.3	2.8	3.3	3.3	3.0	3.5	3.0
				7	2.7	3.3	2.7	3.3	3.0	3.3	3.5
				8	3.3	3.3	3.0	3.8	3.7	3.3	3.0
				9	3.0	3.5	3.3	2.5	3.3	3.0	3.0
				10	2.7	2.5	2.7	2.8	3.0	3.0	3.5
				Mean	3.0†	3.0	3.0†	3.0	3.2†	3.2†	3.1†
				SEM	0.1	0.1	0.1	0.2	0.1	0.1	0.1
3	PT#1223046 (SV-989) GPS18169-002	IP	10 mg/kg QD x 12 wks	1	3.3	3.0	3.7	2.8	3.7	2.8	3.0
				2	2.7	3.3	2.3	3.3	2.7	3.0	2.5
				3	3.0	3.3	3.3	3.0	3.3	3.5	3.5
				4	2.7	3.3	2.7	3.3	3.3	3.3	3.5
				5	2.7	2.8	3.7	2.8	3.0	2.8	3.0
				6	3.0	2.8	2.7	3.0	3.0	3.5	3.0
				7	2.7	2.5	2.7	2.8	2.7	3.3	2.5
				8	3.3	2.5	3.0	3.8	3.3	2.8	2.5
				9	3.0	2.8	2.7	3.5	2.7	3.0	3.5
				10	2.7	3.5	3.0	2.8	2.7	3.5	2.5
				Mean	2.9	3.0	3.0	3.1	3.0	3.2	3.0
				SEM	0.1	0.1	0.1	0.1	0.1	0.1	0.1
4	PT#1223046 (SV-989) GPS18169-002	IP	5 mg/kg QD x 12 wks	1	3.3	3.3	2.7	3.3	2.7	3.5	2.5
				2	2.7	3.5	3.0	4.3	3.3	3.0	3.0
				3	3.0	2.5	2.7	2.5	3.0	2.8	3.5
				4	2.7	3.0	3.0	3.8	3.0	3.3	2.5
				5	2.7	2.8	3.0	2.5	3.3	3.3	3.0
				6	3.7	2.5	3.3	3.0	3.7	2.8	2.5
				7	2.7	2.8	2.7	2.5	3.0	2.8	3.0
				8	3.0	2.5	3.0	2.8	3.3	3.0	3.0
				9	3.7	2.5	2.7	2.5	2.7	2.8	3.5
				10	3.0	2.5	3.0	2.8	3.3	2.8	3.0
				Mean	3.1	2.8	2.9	3.0	3.1	3.0	3.0
				SEM	0.1	0.1	0.1	0.2	0.1	0.1	0.1

Table S5: Water intake in high-fat diet-induced obesity model in C57BL/6 mice during treatment with GPS18169. Individual data.

Gr.	Treatment	Route	Dose	No.	Water Intake (mL/day)							
					Day 57	Day 61	Day 64	Day 68	Day 71	Day 75	Day 78	Day 82
1	Vehicle (Normal Diet)	IP	10 mL/kg QD x 12 wks	1	4.8	5.3	4.8	5.7	5.8	4.3	5.3	5.0
				2	4.3	4.7	5.0	4.7	3.8	4.3	3.8	4.3
				3	4.0	4.7	5.3	5.0	4.3	4.3	4.5	5.0
				4	4.0	5.0	4.0	5.3	4.5	4.0	4.0	5.0
				5	5.0	5.7	6.5	6.0	4.8	4.7	5.0	4.7
				6	4.5	3.7	5.3	5.7	4.0	4.3	4.5	4.0
				7	3.8	3.7	4.8	4.7	4.0	4.3	4.5	4.3
				8	4.3	4.3	5.5	4.7	4.0	3.7	4.8	4.3
				9	4.0	4.3	4.3	4.7	3.5	4.0	4.0	4.0
				10	4.8	5.7	6.0	7.0	4.8	4.7	5.5	4.3
				Mean	4.3	4.7	5.2	5.4	4.4	4.3	4.6	4.5
				SEM	0.1	0.2	0.2	0.2	0.2	0.1	0.2	0.1
2	Vehicle (High-fat Diet)	IP	10 mL/kg QD x 12 wks	1	3.8	2.7	4.5	4.3	4.0	3.7	3.3	3.0
				2	2.8	3.0	3.8	3.7	3.8	3.0	3.3	2.7
				3	3.5	2.7	4.0	3.3	3.8	2.7	2.8	2.7
				4	3.3	3.0	4.0	4.3	3.3	3.0	3.3	3.0
				5	3.0	3.0	4.0	3.0	3.3	2.7	3.3	2.7
				6	6.3	3.3	4.5	4.0	4.0	4.7	4.3	4.7
				7	3.0	3.0	4.0	3.3	3.3	3.3	3.3	2.7
				8	3.3	2.7	4.0	3.3	3.0	3.0	3.0	2.7
				9	2.8	3.3	3.5	3.3	3.3	3.3	3.3	3.3
				10	3.3	3.3	4.3	4.0	2.8	3.3	3.3	3.0
				Mean	3.5†	3.0†	4.1†	3.7†	3.5†	3.3†	3.3†	3.1†
				SEM	0.3	0.1	0.1	0.1	0.1	0.2	0.1	0.2
3	PT#1223046 (SV-989) GPS18169-002	IP	10 mg/kg QD x 12 wks	1	3.3	4.0	4.3	4.7	3.5	3.3	2.8	3.0
				2	3.0	3.3	3.5	3.3	3.0	2.7	2.3	3.0
				3	2.8	3.0	3.8	3.7	3.3	3.0	2.5	3.0
				4	2.8	3.3	4.0	4.0	3.3	2.3	2.8	2.7
				5	2.5	4.0	4.0	3.7	3.0	2.7	2.3	2.7
				6	2.8	3.0	3.5	3.7	3.5	3.0	3.0	3.0
				7	2.3	3.0	3.5	3.0	2.8	3.0	2.8	3.0
				8	3.3	3.7	4.3	4.3	3.3	2.3	2.8	2.3
				9	2.8	3.7	4.0	3.3	3.8	3.3	3.0	3.7
				10	2.8	4.3	4.3	3.7	2.5	2.3	2.8	2.3
				Mean	2.8	3.5	3.9	3.7	3.2	2.8	2.7	2.9
				SEM	0.1	0.2	0.1	0.2	0.1	0.1	0.1	0.1
4	PT#1223046 (SV-989) GPS18169-002	IP	5 mg/kg QD x 12 wks	1	3.3	3.3	3.8	3.3	3.3	2.7	3.3	3.0
				2	3.0	3.3	4.0	3.7	2.8	2.3	2.5	3.0
				3	3.0	3.0	4.0	3.3	3.0	3.0	2.3	2.7
				4	2.8	3.7	4.5	3.7	2.8	2.3	2.3	2.7
				5	2.8	3.0	3.5	3.3	2.8	2.7	2.5	2.7
				6	4.3	5.0	6.8	5.0	3.5	3.3	3.0	3.3
				7	3.0	3.7	4.0	3.7	3.5	2.7	2.5	3.0
				8	3.0	4.0	4.5	4.0	3.5	2.7	3.0	3.3
				9	3.3	3.7	4.0	4.7	3.0	2.7	2.5	3.0
				10	3.3	4.0	4.3	3.3	4.3	2.3	3.0	3.3
				Mean	3.2	3.7	4.3	3.8	3.3	2.7	2.7	3.0
				SEM	0.1	0.2	0.3	0.2	0.2	0.1	0.1	0.1

Gr.	Treatment	Route	Dose	No.	Water Intake (mL/day)							
					Day 85	Day 89	Day 92	Day 96	Day 99	Day 103	Day 106	Day 110
1	Vehicle (Normal Diet)	IP	10 mL/kg QD x 12 wks	1	4.8	5.0	5.8	4.3	6.8	5.7	4.0	6.0
				2	3.5	4.3	4.3	4.3	4.3	3.3	3.5	4.3
				3	4.0	4.0	4.5	4.0	5.8	4.3	3.5	4.0
				4	3.8	4.3	4.8	4.3	4.5	4.0	3.3	5.1
				5	4.8	4.7	5.0	4.3	6.3	4.7	4.3	5.6
				6	4.0	3.7	4.5	4.7	5.3	4.0	3.8	4.7
				7	4.0	4.0	4.3	4.0	5.0	4.0	3.8	4.9
				8	3.8	4.0	4.3	4.7	4.8	4.0	4.5	4.9
				9	3.5	4.0	3.8	3.3	5.5	4.0	3.8	4.1
				10	5.5	5.3	4.5	4.0	3.5	4.7	4.3	6.6
				Mean	4.2	4.2	4.6	4.2	5.2	4.3	3.9	5.0
				SEM	0.2	0.1	0.2	0.1	0.3	0.2	0.1	0.3
2	Vehicle (High-fat Diet)	IP	10 mL/kg QD x 12 wks	1	3.3	3.3	4.0	3.7	3.3	3.0	2.5	3.9
				2	3.5	3.0	3.5	3.3	3.3	3.0	3.3	3.9
				3	2.8	3.0	3.0	3.0	3.0	2.7	2.5	3.0
				4	2.8	3.3	2.8	3.3	3.5	2.3	2.8	3.6
				5	2.8	2.7	3.0	3.3	3.0	2.7	2.3	3.4
				6	4.5	4.7	4.5	3.3	5.5	3.7	3.3	4.4
				7	3.3	3.3	3.3	3.7	3.3	2.7	3.0	3.7
				8	2.8	3.3	2.8	3.7	3.0	3.0	3.0	3.7
				9	3.3	3.7	3.5	3.3	3.8	2.7	3.5	3.3
				10	3.0	3.0	3.0	3.7	3.8	2.7	2.8	4.3
				Mean	3.2†	3.3†	3.3†	3.4†	3.5†	2.8†	2.9†	3.7†
				SEM	0.2	0.2	0.2	0.1	0.2	0.1	0.1	0.1
3	PT#1223046 (SV-989) GPS18169-002	IP	10 mg/kg QD x 12 wks	1	3.5	5.0	3.0	3.3	3.8	3.3	3.3	6.1
				2	2.3	3.3	2.3	3.3	2.5	2.7	3.0	3.6
				3	2.5	3.7	3.0	3.3	3.0	3.3	3.0	4.4
				4	2.3	3.3	2.5	3.7	2.8	3.0	3.0	3.3
				5	2.3	3.7	2.5	3.0	2.5	2.3	2.5	3.4
				6	3.3	3.3	3.8	3.7	3.0	3.0	2.8	3.6
				7	2.5	2.7	2.8	3.0	2.8	3.0	3.3	3.9
				8	3.0	4.0	3.0	3.0	3.0	3.0	2.5	3.7
				9	2.8	4.3	3.3	3.3	3.5	3.0	3.0	3.9
				10	3.0	4.7	3.5	3.0	3.3	4.3	3.8	5.0
				Mean	2.7	3.8	3.0	3.3	3.0	3.1	3.0	4.1
				SEM	0.1	0.2	0.1	0.1	0.1	0.2	0.1	0.3
4	PT#1223046 (SV-989) GPS18169-002	IP	5 mg/kg QD x 12 wks	1	2.5	2.7	3.3	3.3	3.5	3.3	4.5	5.3
				2	2.3	4.7	3.3	3.0	3.0	3.0	3.0	4.3
				3	2.5	2.7	3.3	3.0	2.8	2.3	2.3	3.0
				4	2.5	2.7	2.5	3.0	2.8	2.7	3.0	4.3
				5	2.5	3.3	3.0	3.7	3.3	2.7	2.8	4.0
				6	2.8	3.7	3.0	3.7	3.3	3.0	2.8	3.6
				7	2.3	3.0	2.5	3.3	2.8	2.7	2.5	2.9
				8	3.3	2.7	3.3	3.3	3.3	3.0	2.5	3.6
				9	3.0	2.7	3.5	3.0	3.3	3.3	2.3	3.9
				10	3.0	3.3	3.0	3.7	3.3	3.7	3.3	5.0
				Mean	2.7	3.2	3.1	3.3	3.1	3.0	2.9	4.0
				SEM	0.1	0.2	0.1	0.1	0.1	0.1	0.2	0.2

Gr.	Treatment	Route	Dose	No.	Food Intake (g/day)						
					Day 117	Day 120	Day 124	Day 127	Day 131	Day 134	Day 138
1	Vehicle (Normal Diet)	IP	10 mL/kg QD x 12 wks	1	3.7	3.0	3.3	3.5	3.7	3.5	4.0
				2	4.0	3.8	4.3	3.5	3.3	2.8	3.5
				3	3.7	3.0	4.0	3.3	3.3	4.3	3.0
				4	3.7	3.5	4.0	3.5	3.7	3.5	4.0
				5	4.0	4.0	4.7	4.0	4.0	3.3	3.5
				6	3.7	3.3	3.7	3.3	4.0	4.3	3.5
				7	3.3	3.0	3.7	3.5	3.7	3.5	3.0
				8	3.7	3.3	3.3	3.3	3.3	3.5	3.5
				9	4.0	3.0	2.7	2.8	3.7	4.0	4.0
				10	4.0	3.8	3.3	4.0	4.0	4.0	3.5
				Mean	3.8	3.4	3.7	3.4	3.7	3.7	3.6
				SEM	0.1	0.1	0.2	0.1	0.1	0.1	0.1
2	Vehicle (High-fat Diet)	IP	10 mL/kg QD x 12 wks	1	3.3	3.8	3.3	2.8	3.3	3.5	3.0
				2	3.3	3.0	3.0	2.5	3.0	3.0	3.5
				3	2.7	2.5	2.7	2.8	3.0	2.8	2.5
				4	3.3	2.8	3.3	4.0	3.3	2.8	3.0
				5	2.7	2.5	2.7	2.5	3.3	3.3	2.5
				6	3.3	2.8	3.3	3.3	3.0	3.5	3.0
				7	2.7	3.3	2.7	3.3	3.0	3.3	3.5
				8	3.3	3.3	3.0	3.8	3.7	3.3	3.0
				9	3.0	3.5	3.3	2.5	3.3	3.0	3.0
				10	2.7	2.5	2.7	2.8	3.0	3.0	3.5
				Mean	3.0†	3.0	3.0†	3.0	3.2†	3.2†	3.1†
				SEM	0.1	0.1	0.1	0.2	0.1	0.1	0.1
3	PT#1223046 (SV-989) GPS18169-002	IP	10 mg/kg QD x 12 wks	1	3.3	3.0	3.7	2.8	3.7	2.8	3.0
				2	2.7	3.3	2.3	3.3	2.7	3.0	2.5
				3	3.0	3.3	3.3	3.0	3.3	3.5	3.5
				4	2.7	3.3	2.7	3.3	3.3	3.3	3.5
				5	2.7	2.8	3.7	2.8	3.0	2.8	3.0
				6	3.0	2.8	2.7	3.0	3.0	3.5	3.0
				7	2.7	2.5	2.7	2.8	2.7	3.3	2.5
				8	3.3	2.5	3.0	3.8	3.3	2.8	2.5
				9	3.0	2.8	2.7	3.5	2.7	3.0	3.5
				10	2.7	3.5	3.0	2.8	2.7	3.5	2.5
				Mean	2.9	3.0	3.0	3.1	3.0	3.2	3.0
				SEM	0.1	0.1	0.1	0.1	0.1	0.1	0.1
4	PT#1223046 (SV-989) GPS18169-002	IP	5 mg/kg QD x 12 wks	1	3.3	3.3	2.7	3.3	2.7	3.5	2.5
				2	2.7	3.5	3.0	4.3	3.3	3.0	3.0
				3	3.0	2.5	2.7	2.5	3.0	2.8	3.5
				4	2.7	3.0	3.0	3.8	3.0	3.3	2.5
				5	2.7	2.8	3.0	2.5	3.3	3.3	3.0
				6	3.7	2.5	3.3	3.0	3.7	2.8	2.5
				7	2.7	2.8	2.7	2.5	3.0	2.8	3.0
				8	3.0	2.5	3.0	2.8	3.3	3.0	3.0
				9	3.7	2.5	2.7	2.5	2.7	2.8	3.5
				10	3.0	2.5	3.0	2.8	3.3	2.8	3.0
				Mean	3.1	2.8	2.9	3.0	3.1	3.0	3.0
				SEM	0.1	0.1	0.1	0.2	0.1	0.1	0.1

Table S6: Body weight changes in high-fat diet-induced obesity model in C57Bl/6 mice during treatment with GPS18169. Individual data.

Gr.	Treatment	Route	Dose	No.	Body Weight (g) Day 1	Day 8	Day 15	Day 22	Day 29	Day 36	Day 43	Day 50	Day 55	Day 58
1	Vehicle (Normal Diet)	IP	10 mL/kg QD x 12 wks	1	19	22	24	24	25	26	27	27	27	26
				2	21	23	25	25	26	27	27	28	28	27
				3	19	23	25	25	26	27	28	29	30	28
				4	19	22	23	24	25	25	26	27	27	26
				5	20	22	24	25	27	29	29	30	31	30
				6	18	23	24	25	26	27	27	27	28	27
				7	19	23	24	25	26	26	26	27	28	27
				8	19	20	22	23	24	24	25	26	26	26
				9	17	20	22	23	24	24	25	26	26	25
				10	19	23	24	25	26	27	28	29	29	28
				Mean	19.0	22.1	23.7	24.4	25.4	26.1	26.7	27.4	27.9	26.9
				SEM	0.4	0.4	0.3	0.3	0.3	0.5	0.4	0.4	0.5	0.5
2	Vehicle (High-fat Diet)	IP	10 mL/kg QD x 12 wks	1	20	24	26	28	30	33	34	35	36	34
				2	20	24	26	31	35	38	40	42	44	43
				3	18	22	24	28	31	33	35	36	37	36
				4	21	26	29	34	37	41	44	45	47	45
				5	19	21	24	27	30	32	34	35	37	35
				6	20	25	28	33	36	40	42	45	46	45
				7	18	23	24	28	31	34	35	39	40	40
				8	19	25	27	30	33	38	39	41	43	42
				9	20	23	27	32	35	40	41	43	45	44
				10	19	24	27	31	36	38	39	40	41	41
				Mean	19.3	23.7	26.2	30.2	33.4↑	36.7↑	38.3↑	40.1↑	41.6↑	40.5↑
				SEM	0.4	0.5	0.6	0.8	0.9	1.1	1.1	1.2	1.3	1.3
				% of Day 55 (pre-dose)	--	--	--	--	--	--	--	--	100	97
3	PT#1223046 (SV-989) GPS18169-002	IP	10 mg/kg QD x 12 wks	1	21	25	28	33	37	40	43	44	45	45
				2	20	24	25	30	34	38	40	42	44	42
				3	19	23	25	30	32	36	38	41	43	42
				4	21	26	29	33	36	39	42	44	45	44
				5	19	24	26	30	34	38	39	42	43	42
				6	19	23	26	31	34	37	38	38	39	38
				7	18	23	25	29	31	35	36	38	39	38
				8	19	23	26	27	29	32	34	35	37	36
				9	20	25	27	30	33	38	39	41	43	42
				10	18	23	25	29	33	36	37	38	40	39
				Mean	19.8	23.9	26.2	30.2	33.3	36.9	38.6	40.3	41.8	40.8
				SEM	0.4	0.3	0.4	0.6	0.7	0.7	0.8	0.9	0.9	0.9
				% of Day 55 (pre-dose)	--	--	--	--	--	--	--	--	100	98
				Decrease % vs. Gr. 2	--	--	--	--	--	--	--	--	0.0	-0.3
4	PT#1223046 (SV-989) GPS18169-002	IP	5 mg/kg QD x 12 wks	1	19	23	25	29	32	34	36	37	38	37
				2	21	26	28	32	35	38	39	39	40	40
				3	19	25	28	33	36	40	40	42	43	42
				4	19	22	25	27	30	34	34	35	36	35
				5	18	24	27	32	36	40	42	44	46	45
				6	20	24	26	30	34	37	38	39	41	39
				7	20	23	26	28	30	33	34	36	38	37
				8	20	23	25	27	31	34	37	38	40	39
				9	20	24	26	31	34	37	39	41	42	41
				10	19	23	25	28	30	33	35	37	38	37
				Mean	19.0	23.7	26.1	29.7	33.1	36.3	37.7	39.0	40.4	39.4
				SEM	0.2	0.4	0.4	0.7	0.8	0.9	0.9	0.9	1.0	1.0
				% of Day 55 (pre-dose)	--	--	--	--	--	--	--	--	100	98
				Decrease % vs. Gr. 2	--	--	--	--	--	--	--	--	0.0	-0.2

Gr.	Treatment	Route	Dose	No.	Body Weight (g)								
					Day 61	Day 63	Day 65	Day 68	Day 70	Day 72	Day 75	Day 77	Day 79
1	Vehicle (Normal Diet)	IP	10 mL/kg QD x 12 wks	1	27	26	26	27	27	27	27	27	28
				2	28	28	28	28	29	29	29	29	29
				3	29	29	29	29	30	30	29	30	31
				4	27	27	27	27	28	27	27	28	28
				5	30	30	30	31	32	31	30	31	31
				6	28	28	28	29	30	29	30	30	31
				7	27	28	28	28	28	28	28	28	29
				8	26	26	27	27	28	28	27	28	28
				9	26	26	26	26	27	27	26	26	26
				10	28	28	28	29	30	31	30	30	30
				Mean	27.6	27.6	27.7	28.1	28.8	28.4	28.1	28.6	29.0
				SEM	0.4	0.4	0.4	0.5	0.5	0.5	0.5	0.5	0.5
2	Vehicle (High-fat Diet)	IP	10 mL/kg QD x 12 wks	1	35	36	37	38	38	39	40	41	42
				2	44	44	44	44	45	45	45	46	46
				3	37	37	37	38	38	39	40	41	42
				4	46	47	47	47	48	48	48	49	49
				5	36	36	37	36	36	37	37	37	38
				6	46	46	47	47	47	48	49	50	50
				7	41	41	42	42	43	44	45	46	46
				8	43	43	44	44	45	45	45	46	46
				9	45	45	45	45	46	47	47	48	48
				10	43	43	44	45	45	46	47	48	48
				Mean	41.6†	41.8†	42.4†	42.6†	43.1†	43.8†	44.3†	45.2†	45.5†
				SEM	1.3	1.3	1.3	1.2	1.3	1.3	1.3	1.3	1.2
				% of Day 55 (pre-dose)	100	100	102	102	104	105	106	109	109
3	PT#1223046 (SV-989) GPS18169-002		10 mg/kg QD x 12 wks	1	46	47	47	47	48	48	48	49	48
				2	42	43	43	43	44	44	45	44	44
				3	43	43	43	43	44	44	44	43	43
				4	44	44	44	44	45	45	45	46	47
				5	43	43	44	44	45	45	46	46	46
				6	39	39	40	40	40	41	42	43	42
				7	39	39	39	39	40	39	39	38	37
				8	37	37	38	38	39	39	39	40	40
				9	43	43	43	43	44	45	46	47	47
				10	39	39	40	40	40	40	39	38	37
				Mean	41.5	41.7	42.1	42.1	42.9	43.0	43.3	43.4	43.1
				SEM	0.9	1.0	0.9	0.9	0.9	1.0	1.1	1.2	1.3
				% of Day 55 (pre-dose)	99	100	101	101	103	103	104	104	103
				Decrease % vs. Gr. 2	0.7	0.7	1.2	1.6	0.9	2.3	2.7	4.4	5.7
4	PT#1223046 (SV-989) GPS18169-002	IP	5 mg/kg QD x 12 wks	1	37	38	38	38	39	39	38	37	37
				2	42	42	43	43	43	42	42	41	41
				3	42	42	43	43	43	44	44	45	45
				4	35	36	36	36	37	38	37	36	35
				5	46	45	45	45	46	47	47	47	47
				6	40	40	40	40	40	41	41	42	43
				7	37	38	38	38	39	39	40	41	41
				8	39	39	40	40	40	41	42	43	42
				9	42	42	42	43	44	44	44	44	44
				10	38	39	39	38	38	38	39	40	39
				Mean	39.8	40.1	40.4	40.4	41.2	41.7	41.7	41.8	41.7
				SEM	1.0	0.8	0.9	0.9	0.9	0.9	1.0	1.1	1.2
				% of Day 55 (pre-dose)	99	99	100	100	102	103	103	103	103
				Decrease % vs. Gr. 2	1.5	1.2	1.9	2.3	1.6	2.0	3.1	4.8	5.6

Gr.	Treatment	Route	Dose	No.	Body Weight (g)								
					Day 82	Day 84	Day 86	Day 89	Day 91	Day 93	Day 96	Day 98	Day 100
1	Vehicle (Normal Diet)	IP	10 mL/kg QD x 12 wks	1	27	28	28	29	28	28	29	28	28
				2	30	30	31	31	31	30	31	31	31
				3	30	30	31	31	31	31	31	31	32
				4	28	28	28	28	28	28	28	27	26
				5	31	32	32	32	32	32	32	31	32
				6	31	31	31	31	31	31	32	31	31
				7	28	29	29	29	29	28	29	28	29
				8	28	28	29	28	28	28	29	28	29
				9	26	27	27	27	27	27	28	27	28
				10	30	30	30	30	30	30	30	30	31
				Mean	28.8	29.3	29.6	29.6	29.5	29.2	29.9	29.1	29.6
				SEM	0.6	0.5	0.5	0.5	0.5	0.6	0.5	0.6	0.7
2	Vehicle (High-fat Diet)	IP	10 mL/kg QD x 12 wks	1	42	42	42	42	41	40	39	38	38
				2	46	46	47	47	48	48	48	48	49
				3	42	43	44	45	46	45	46	45	46
				4	49	49	50	49	49	49	49	49	49
				5	38	38	38	38	37	37	37	36	36
				6	51	50	51	52	53	52	51	50	50
				7	47	47	48	49	49	49	49	49	49
				8	47	47	48	48	48	48	49	49	48
				9	49	50	50	51	52	52	52	52	52
				10	49	49	50	50	50	50	51	50	50
				Mean	46.0†	46.1†	46.8†	47.1†	47.3†	47.0†	47.1†	46.6†	46.7†
				SEM	1.3	1.3	1.3	1.4	1.5	1.6	1.6	1.7	1.7
				% of Day 55 (pre-dose)	111	111	113	113	114	113	113	112	112
3	PT#1223046 (SV-989) GPS18169-002	IP	10 mg/kg QD x 12 wks	1	48	49	49	49	49	49	49	49	49
				2	43	43	42	42	42	41	41	40	41
				3	42	41	40	39	38	38	37	36	35
				4	48	47	48	48	47	48	48	48	47
				5	46	47	48	48	48	47	47	47	46
				6	43	44	44	44	45	44	45	45	45
				7	36	36	35	35	34	33	32	32	31
				8	40	41	41	41	42	42	43	44	44
				9	47	47	47	46	47	47	48	48	48
				10	36	35	35	34	33	32	31	30	29
				Mean	42.9	43.0	42.9	42.6	42.5	42.1	42.1	41.9	41.5
				SEM	1.4	1.5	1.6	1.7	1.8	1.9	2.1	2.2	2.3
				% of Day 55 (pre-dose)	103	103	103	102	102	101	101	100	99
				Decrease % vs. Gr. 2	7.2	7.2	8.8	10.0	10.6	10.9	11.0	10.5	11.6
4	PT#1223046 (SV-989) GPS18169-002	IP	5 mg/kg QD x 12 wks	1	36	35	34	33	32	31	31	30	29
				2	40	40	39	38	37	37	36	35	34
				3	45	45	45	45	44	43	44	44	44
				4	34	34	33	32	32	31	30	30	29
				5	48	48	49	48	49	49	49	49	49
				6	42	43	42	42	41	41	42	41	40
				7	42	42	43	44	45	46	46	46	46
				8	43	44	45	46	47	46	46	47	47
				9	45	45	45	46	45	45	46	46	46
				10	39	38	37	36	36	35	35	34	33
				Mean	41.7	41.4	41.2	41.0	40.8	40.4	40.5	40.2	39.7
				SEM	1.4	1.4	1.7	1.8	2.0	2.1	2.2	2.3	2.5
				% of Day 55 (pre-dose)	103	102	102	101	101	100	100	100	98
				Decrease % vs. Gr. 2	6.7	7.5	9.4	10.4	11.2	11.5	11.5	11.2	12.5

Gr.	Treatment	Route	Dose	No.	Body Weight (g)								
					Day 103	Day 105	Day 107	Day 110	Day 112	Day 114	Day 117	Day 119	Day 121
1	Vehicle (Normal Diet)	IP	10 mL/kg QD x 12 wks	1	29	29	30	29	29	28	29	29	30
				2	30	31	30	31	31	30	31	32	32
				3	32	32	32	33	33	32	33	33	33
				4	25	25	26	27	27	27	28	29	30
				5	32	31	32	32	32	31	32	33	34
				6	30	31	31	32	32	31	33	34	33
				7	29	29	28	28	29	28	29	29	29
				8	29	29	29	30	29	28	29	30	30
				9	27	27	27	27	28	27	28	29	29
				10	31	31	30	30	31	30	31	32	31
				Mean	29.2	29.3	29.5	29.9	30.1	29.2	30.2	30.9	31.1
				SEM	0.7	0.7	0.6	0.7	0.6	0.6	0.6	0.7	0.6
2	Vehicle (High-fat Diet)	IP	10 mL/kg QD x 12 wks	1	37	36	36	35	35	34	33	32	33
				2	48	48	49	48	48	47	48	49	49
				3	46	45	45	44	44	43	43	43	42
				4	49	48	47	46	47	45	46	46	45
				5	35	35	34	33	33	32	33	33	33
				6	49	48	47	46	46	45	47	48	49
				7	48	49	48	48	48	47	48	48	47
				8	48	47	46	46	47	45	46	47	47
				9	51	51	51	50	50	49	51	52	53
				10	49	50	50	49	49	48	49	49	48
				Mean	46.0†	45.7†	45.3†	44.5†	44.7†	43.5†	44.4†	44.7†	44.6†
				SEM	1.7	1.8	1.8	1.8	1.9	1.8	2.0	2.2	2.1
				% of Day 55 (pre-dose)	111	110	109	107	107	105	107	107	107
3	PT#1223046 (SV-989) GPS18169-002	IP	10 mg/kg QD x 12 wks	1	49	48	49	48	48	47	46	45	45
				2	40	40	40	39	38	37	36	35	34
				3	34	34	33	32	32	31	31	31	30
				4	46	46	47	47	47	47	46	45	44
				5	45	44	45	45	46	44	43	42	41
				6	46	45	46	46	46	45	45	44	43
				7	31	30	31	30	31	29	30	29	28
				8	43	42	42	41	41	40	39	38	37
				9	47	46	46	46	46	45	44	43	42
				10	29	28	28	27	27	26	26	26	26
				Mean	41.0	40.3	40.7	40.1	40.2	39.1	38.6	37.8	37.0*
				SEM	2.3	2.3	2.4	2.5	2.4	2.5	2.3	2.3	2.2
				% of Day 55 (pre-dose)	98	96	97	96	96	94	92	90	89
				Decrease % vs. Gr. 2	11.3	12.2	10.6	10.3	10.5	10.5	13.5	15.8	17.4
4	PT#1223046 (SV-989) GPS18169-002	IP	5 mg/kg QD x 12 wks	1	28	28	28	28	28	27	27	27	27
				2	33	32	31	30	30	29	29	28	29
				3	43	43	43	42	42	41	41	41	40
				4	28	27	27	27	27	26	26	26	26
				5	49	50	50	51	51	50	50	50	51
				6	39	38	37	36	36	35	34	34	33
				7	46	46	46	46	45	44	45	45	44
				8	47	46	45	44	44	43	41	40	39
				9	47	47	46	45	45	43	41	40	38
				10	33	33	32	32	31	30	30	29	29
				Mean	39.3	39.0	38.5	38.1	37.9	36.8	37.1*	36.8*	36.3*
				SEM	2.6	2.7	2.7	2.7	2.7	2.7	2.7	2.7	2.6
				% of Day 55 (pre-dose)	97	97	95	94	94	91	92	91	90
				Decrease % vs. Gr. 2	12.0	12.1	12.5	11.8	12.7	12.9	14.0	15.2	16.2

Gr.	Treatment	Route	Dose	No.	Body Weight (g)								
					Day 124	Day 126	Day 128	Day 131	Day 133	Day 135	Day 138	Day 140	Day 141
1	Vehicle (Normal Diet)	IP	10 mL/kg QD x 12 wks	1	29	30	30	30	30	30	30	29	26
				2	31	31	30	30	30	29	30	30	26
				3	33	33	33	33	33	32	33	33	29
				4	30	31	31	30	30	31	32	31	28
				5	35	35	34	34	34	34	33	33	29
				6	34	34	34	34	34	33	34	34	29
				7	29	30	30	30	30	30	29	30	27
				8	30	30	30	30	30	30	31	30	26
				9	30	30	29	28	28	28	28	28	25
				10	32	33	33	33	33	33	33	33	29
				Mean	31.2	31.6	31.2	31.0	31.2	31.0	31.3	30.9	27.2
				SEM	0.7	0.6	0.6	0.7	0.7	0.6	0.6	0.6	0.5
2	Vehicle (High-fat Diet)	IP	10 mL/kg QD x 12 wks	1	34	35	34	34	33	33	32	33	30
				2	50	50	51	52	51	52	53	52	48
				3	41	40	40	39	38	37	36	36	33
				4	45	44	44	43	43	42	42	42	39
				5	32	32	32	31	31	30	30	29	27
				6	51	52	53	54	54	54	53	54	51
				7	47	46	46	45	45	44	44	44	40
				8	47	47	46	46	45	45	44	44	41
				9	53	54	55	54	55	56	56	55	51
				10	47	46	45	44	43	43	43	43	40
				Mean	44.7†	44.6†	44.6†	44.2†	43.8†	43.6†	43.3†	43.2†	40.0†
				SEM	2.2	2.2	2.4	2.5	2.6	2.7	2.8	2.8	2.6
				% of Day 55 (pre-dose)	107	107	107	106	105	105	104	104	96
3	PT#1223046 (SV-989) GPS18169-002	IP	10 mg/kg QD x 12 wks	1	45	44	44	44	44	44	43	43	39
				2	33	32	32	31	30	30	29	29	26
				3	30	29	30	29	30	30	30	29	26
				4	43	42	42	41	40	39	38	37	33
				5	40	40	39	38	37	37	36	35	32
				6	42	41	40	39	38	38	37	36	33
				7	28	28	28	28	28	28	29	28	25
				8	36	35	34	34	33	33	33	33	29
				9	41	39	38	36	35	35	34	34	31
				10	27	27	27	26	26	26	27	27	25
				Mean	36.5*	35.7*	35.4*	34.6*	34.1*	34.0*	33.6*	33.1*	29.9*
				SEM	2.1	2.0	1.9	1.9	1.8	1.8	1.6	1.6	1.4
				% of Day 55 (pre-dose)	87	85	85	83	82	81	80	79	72
				Decrease % vs. Gr. 2	18.7	20.3	21.0	22.1	22.5	22.4	22.8	23.7	25.6
4	PT#1223046 (SV-989) GPS18169-002	IP	5 mg/kg QD x 12 wks	1	26	26	26	26	26	26	26	26	24
				2	29	30	30	29	29	30	30	29	26
				3	40	39	38	37	36	35	34	34	31
				4	27	26	26	26	26	26	26	25	23
				5	52	52	52	51	51	51	51	51	47
				6	32	31	31	31	30	29	28	28	25
				7	43	42	42	41	41	42	41	40	36
				8	38	37	36	34	33	32	32	32	28
				9	37	36	35	34	33	33	32	31	28
				10	29	28	29	29	29	29	30	29	26
				Mean	36.0*	35.4*	35.1*	34.3*	33.4*	33.3*	33.0*	32.9*	29.8*
				SEM	2.7	2.6	2.6	2.5	2.4	2.5	2.4	2.6	2.4
				% of Day 55 (pre-dose)	89	88	87	85	83	82	82	81	74
				Decrease % vs. Gr. 2	17.1	18.3	19.0	20.1	21.5	21.4	21.5	21.6	23.3
